# An Update in Qualitative Imaging of Bone Using Ultrashort Echo Time Magnetic Resonance

**DOI:** 10.3389/fendo.2020.555756

**Published:** 2020-09-29

**Authors:** Saeed Jerban, Douglas G. Chang, Yajun Ma, Hyungseok Jang, Eric Y. Chang, Jiang Du

**Affiliations:** ^1^Department of Radiology, University of California, San Diego, San Diego, CA, United States; ^2^Departments of Orthopaedic Surgery, University of California, San Diego, San Diego, CA, United States; ^3^Research Service, Veterans Affairs San Diego Healthcare System, San Diego, CA, United States

**Keywords:** cortical bone, trabecular bone, MRI, UTE–ultra-short TE, single inversion recovery UTE, zero echo time MRI

## Abstract

Bone is comprised of mineral, collagenous organic matrix, and water. X-ray-based techniques are the standard approach for bone evaluation in clinics, but they are unable to detect the organic matrix and water components in bone. Magnetic resonance imaging (MRI) is being used increasingly for bone evaluation. While MRI can non-invasively assess the proton pools in soft tissues, cortical bone typically appears as a signal void with clinical MR techniques because of its short T2*. New MRI techniques have been recently developed to image bone while avoiding the ionizing radiation present in x-ray-based methods. Qualitative bone imaging can be achieved using ultrashort echo time (UTE), single inversion recovery UTE (IR-UTE), dual-inversion recovery UTE (Dual-IR-UTE), double-inversion recovery UTE (Double-IR-UTE), and zero echo time (ZTE) sequences. The contrast mechanisms as well as the advantages and disadvantages of each technique are discussed.

## Background

Osteoporosis (OP) is a bone disease which affects millions of people around the world (1) and can lead to serious long-term disability in many patients. OP development always occurs in synchrony with increases in cortical bone porosity and with trabecular bone deterioration (2). The development of non-invasive imaging techniques to evaluate bone structural properties and stability is crucial to improved diagnosis of OP and monitoring of OP patients undergoing medical treatments.

Bone is a highly complex hierarchical structure (3) of organic matrix combined with hydroxyapatite calcium phosphate (HA) crystals. From the architectural point of view, cortical bone (compact) and trabecular bone (spongy) are two main morphologies of the bone tissue with approximate porosities under 20% and over 80%, respectively (2, 4). Cortical bone comprises around 80% of human bone mass (5, 6). Trabecular bone generally exists surrounded by cortical bone near joints. Despite large pores in trabecular bone sites, most cortical porosities are limited to pores below 100 µm in size (7, 8).

Water in cortical and trabecular bone exist in different states and at various locations (7, 9). In healthy bone, the main portion of water exists in “bound” form to HA crystals and to the collagenous matrix (6, 8, 10). The remaining water volume in bone resides in pores ranging from sub-microns to hundreds of micrometers in size (7, 8). Bound water indicates the bone mineral and collagenous matrix, while pore water indicates the porosity of bone (11, 12). Average bound water T2* is approximately 300 µs whereas pore water T2* is longer than 1,000 µs and can reach up to several milliseconds (10, 13–15). Collagen protons have extremely short T2*s, on the order of several microseconds (10).

Bone mineral density (BMD) measurement has been the standard bone evaluation method in clinics performed using x-ray-based techniques including dual-energy x-ray absorptiometry (DEXA) and quantitative computed tomography (QCT) (2, 16, 17). DEXA-based measurement of BMD is non-reliable due to very low resolution and its 2D nature. BMD as a predictive clinical measurement is quite limited in its representation of bone microstructure and, consequently, of bone fragility, functionality, and fracture risk (18–21). However, these non-mineral components may describe the bone microstructural and biomechanical properties independently from BMD. Although, QCT enables the measurement of bone microstructure in addition to BMD however it comes with a high radiation dose.

Employing magnetic resonance imaging (MRI) for bone evaluation has been increasingly reported in the literature. MRI-based techniques for bone evaluation avoid the potential harm associated with x-ray-based imaging techniques (5, 6, 16, 22). MRI-based bone evaluation can also provide valuable evaluation of the surrounding soft tissues including tendons (23) and muscles, advantages that are not available in x-ray-based techniques. Bone has a short apparent transverse relaxation time (T2*) and is typically visualized with a void signal when using conventional clinical pulse sequences with echo times (TEs) of a several milliseconds or longer (24, 25). The lack of direct signal originating from bone impairs the ability of conventional MRI sequences to provide any qualitative or quantitative bone assessments. It should be noted that MRI has been used in the past to measure bone microstructure *via* indirect visualization of the dark regions (bone) in high-resolution conventional acquisitions, however, this approach is limited to distal bone sites and is very motion-sensitive (9, 26). Recently, new MRI techniques such as ultrashort echo time (UTE)-MRI have been developed for direct bone imaging and associated quantitative measurements (5, 6, 16, 22).

Qualitative bone imaging can be achieved using conventional, ultrashort echo time (UTE), adiabatic inversion recovery UTE (IR-UTE), dual-inversion recovery UTE (Dual-IR-UTE), double-inversion recovery UTE (Double-IR-UTE), UTE with rescaled echo subtraction (UTE-RS), Fat suppression UTE, Water- and fat-suppressed proton projection imaging (WASPI), and zero echo time (ZTE) sequences. The contrast mechanisms as well as the advantages and disadvantages of each technique are discussed in detail. A brief comparison between discussed MRI techniques is presented in **Table 1**. This review will be an update to our previously published review paper in 2013 (6). As UTE-MRI bone imaging field is experiencing fast growth, it is believed that revisiting this review topic would be benedictional to bone imaging society.

**Table 1:** Comparing qualitative MRI techniques for bone imaging.

MRI technique	Relative bone signal	Visualized proton pool	Contrast	Cortical or trabecular bone	B0 sensitivity	B1 sensitivity	Scan time-efficiency*	Appropriate for axial bone sites (e.g., spine, pelvis)
Conventional FSE (27)	Very Low	Water in large pores	High (reverse contrast)	Partial cortical bone	Insensitive	Insensitive	High	High
Conventional STE (28)	Very Low	Water in large pores	High (reverse contrast)	Partial cortical bone	Sensitive	Insensitive	High	High
Basic UTE (6, 8, 10, 13, 15, 29)	High	Bound and pore water	Low	Cortical bone	Insensitive	Insensitive	Relatively high	High
IR-UTE (25, 30–39)	High	Bound water	High	Cortical and trabecular bone	Insensitive	Insensitive	Low	High
Dual-IR UTE (33, 40, 41)	High	Bound water	High	Cortical and trabecular bone	Sensitive	Insensitive	Low	Moderate
Double-IR UTE (42)	High	Bound water	High	Cortical and trabecular bone	Insensitive	Insensitive	Low	High
Fat suppression UTE (43–45)	High	Bound and pore water	Moderate	Cortical and trabecular bone	Sensitive	Insensitive	Moderate	Moderate
UTE-RS (46, 47)	High	Bound and pore water	High	Cortical bone	Sensitive	Insensitive	Moderate	Moderate
WASPI (48–51)	High	Bound water	High	Cortical and trabecular bone	Sensitive	Sensitive	Relatively low	Moderate
ZTE (52–56)	Moderate (low flip angle)	Bound and pore water	Low	Cortical bone	Insensitive	Insensitive	Relatively high	High

## UTE and IR-UTE Pulse Sequences and their Contrast Mechanism

**Figure 1:**
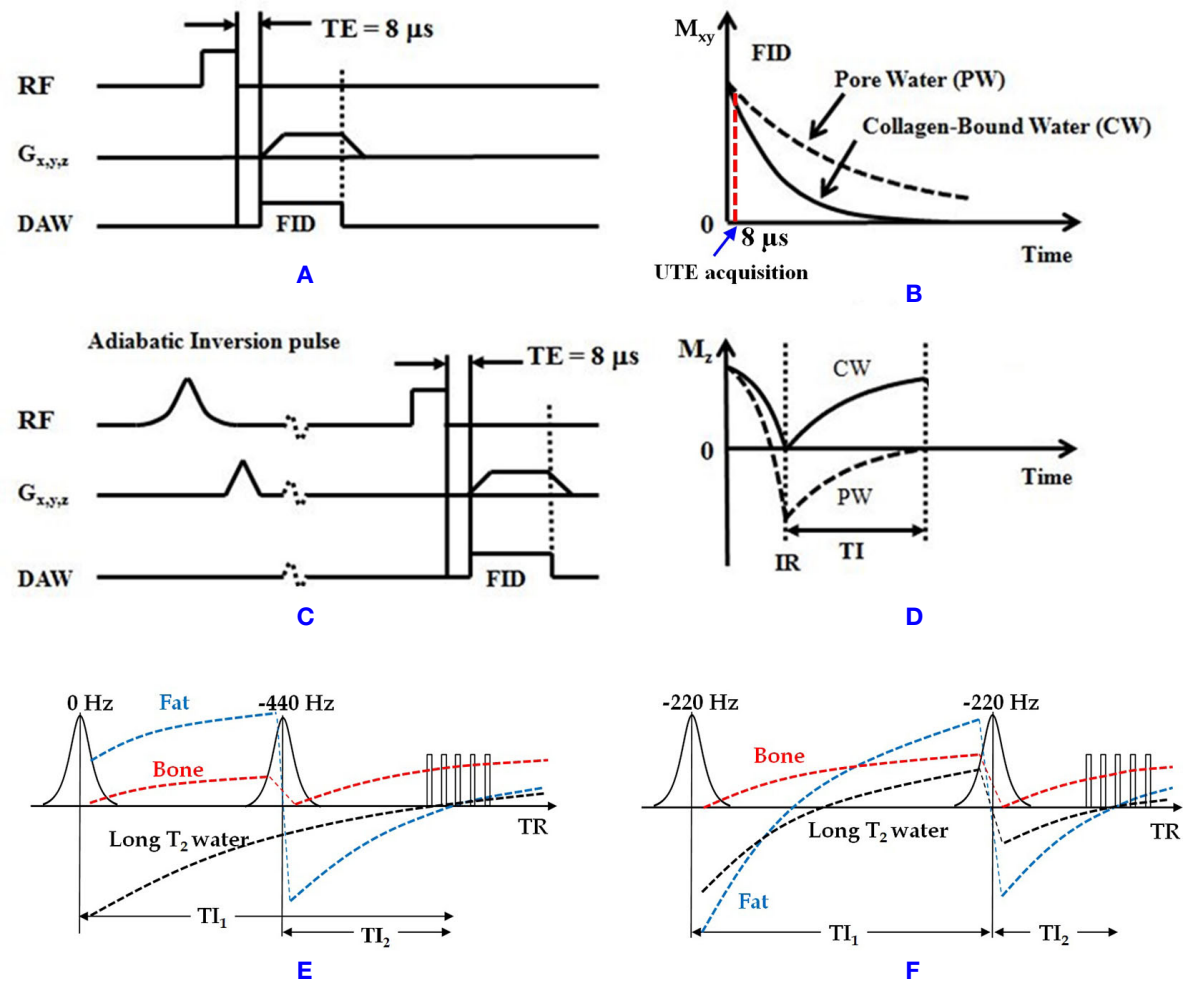
**(A)** The 3D UTE and **(C)** IR-UTE sequences, as well as the contrast mechanisms for imaging of **(B)** total water and **(D)** collagen-bound water. The UTE sequence employs a short rectangular pulse for signal excitation followed by 3D radial ramp sampling with a minimal nominal TE of 8 µs. The schematic time point for UTE acquisition at TE of 8 µs is shown in B. The IR-UTE sequence employs an adiabatic inversion pulse to invert and null the pore water magnetization. The collagen-bound water magnetization is not inverted and is detected by the subsequent UTE data acquisition. For 2D UTE and IR-UTE imaging the rectangular pulse is replaced with a half RF pulse for slice selective excitation. **(E)** Dual-IR and **(F)** Double-IR UTE sequences combined with the contrast mechanism for bone imaging (bound water). The Dual-IR UTE sequence utilizes two adiabatic IR pulses with center frequencies of 0 and −440 Hz with narrow bandwidth of around 500 Hz (fat frequency offset at 3T) to invert long T_2_ water and fat, respectively. The Double-IR UTE sequence utilizes two identical adiabatic IR pulses with a center frequency of −220 Hz with broad bandwidth of no less than 1000 Hz (half of the fat frequency offset at 3T) to invert both long T_2_ water and fat. Bone signal is largely saturated by each adiabatic IR pulse due to its fast T_2_* relaxation during the inversion period. At the optimized TI_1_ and TI_2_ in both Dual- and Double-IR UTE sequences, both long T_2_ water and fat can be suppressed simultaneously, and the bone signals can be acquired with multiple UTE spokes This figure was partially presented before by Ma et al. (42). Reprinting permission is granted through Rightslink system. The figure is modified for presentation purposes. Minor modifications were performed for presentation purposes. RF, radio frequency pulse; TE, echo time; G_x,y,z_, gradient magnetic field in X, Y, Z directions; DAW, data acquisition window; M_z_, longitudinal magnetization; M_xy_, transverse magnetization; CW, collagen-bound water; PW, pore water; IR, inversion recovery; TI, inversion time.

Both UTE and IR-UTE sequences have been developed for imaging of cortical and trabecular bone as described in the following sections. Representative 3D UTE and IR-UTE sequences are shown in **Figure 1**. The basic 3D UTE sequence (**Figure 1A**) employs a short radiofrequency (RF) rectangular pulse (duration = 26–52 µs) for signal excitation followed by 3D radial ramp sampling with minimal nominal TEs of 8 to 50 µs depending on the hardware. For 3D UTE Cones sequence, sampling is performed over a k-space divided into multiple cone shapes with twisted radial trajectories along each cone. The Cones trajectories are more time-efficient than radial trajectories in covering 3D k-space (46), and resolve the limitations associated with 2D UTE sequences, namely, sensitivity to eddy currents (57). Furthermore, the 3D UTE Cones sequence allows anisotropic fields of view and spatial resolution, resulting in reduced scan times (58–60). For 2D UTE imaging the rectangular pulse is replaced with a half RF pulse for slice selective excitation. Both bound water and pore water can be detected using the basic UTE sequences (**Figure 1B**) (14). Adiabatic inversion recovery prepared UTE (IR-UTE) sequences have been developed for selective imaging of collagen-bound water (**Figure 1C**). With the IR-UTE sequence, a Silver-Hoult adiabatic inversion pulse (duration = 8.64 ms) is used to invert the pore water longitudinal magnetization. The longitudinal magnetization of bound water with a very short T2^*^ cannot be inverted, but can be largely saturated, by the adiabatic IR pulse due to major relaxation of bound water magnetization during the adiabatic inversion pulse. The UTE acquisition is initiated to selectively detect signal from bound water only after an inversion time (TI) when the inverted pore water magnetization approaches the null point (**Figure 1D**).

## Conventional MR for Bone Imaging

**Figure 2:**
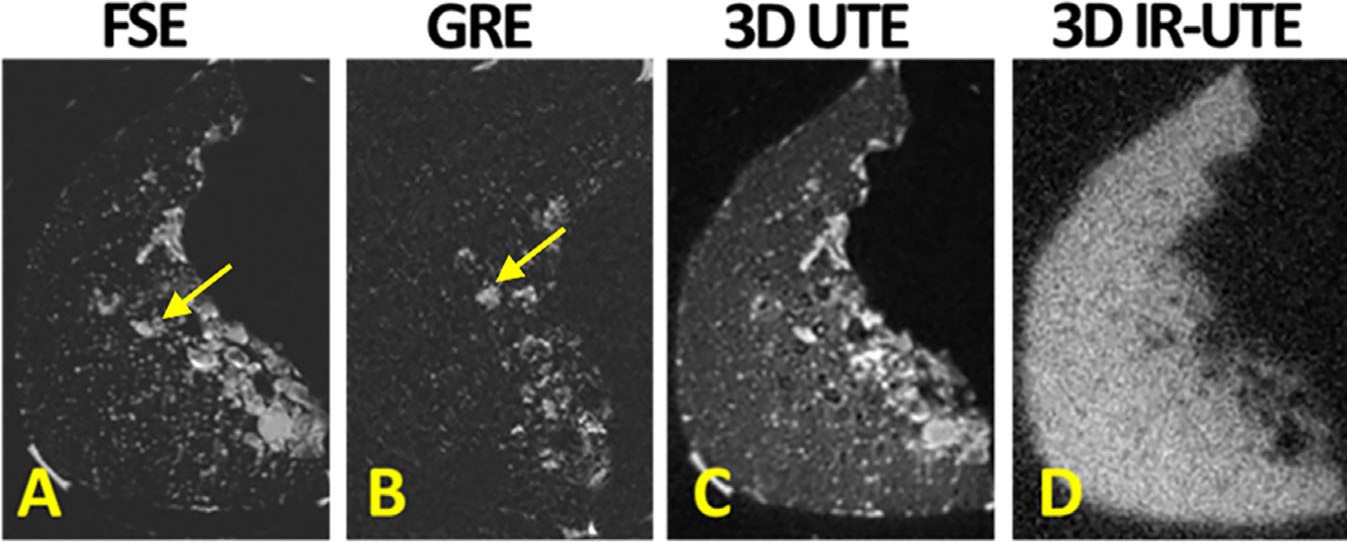
**(A)** Conventional fast spin echo (FSE), **(B)** gradient echo (GRE), **(C)** 3D ultrashort echo time (3D UTE), **(D)** 3D adiabatic inversion recovery UTE (3D IR-UTE) MRI images in axial plane of a piece of human cortical bone harvested from the anterior tibial midshaft. FSE and GRE only detect signal from pore water, likely in Haversian canals (indicated with yellow arrow). UTE MRI results in high signal from all sites of the bone specimen. IR-UTE detects signal from bone with much higher contrast than UTE MRI can. his figure was previously presented by Du et al. (13). Reprinting permission is granted through Rightslink system. The figure is modified for presentation purposes. Minor modifications were performed for presentation purposes.

**Figure 3:**
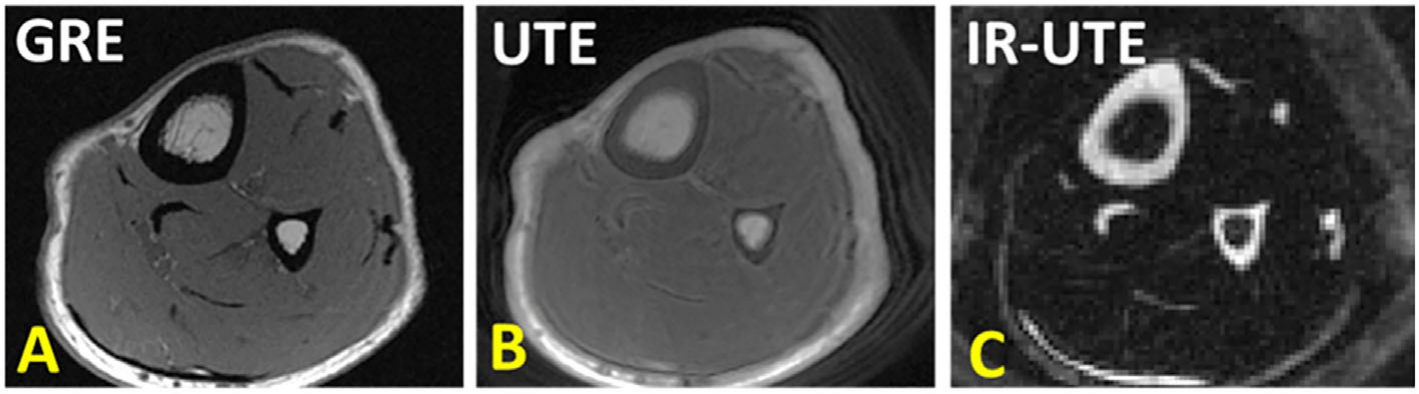
**(A)** Conventional gradient echo (GRE), **(B)** ultrashort echo time (UTE), and **(C)** adiabatic inversion recovery UTE (IR-UTE) image in axial plane of the lower leg. GRE results in void signal in bone. UTE MRI results in high signal for bone, but low contrast. IR-UTE results in higher contrast between cortical bone and surrounding soft tissue, similar to that can be seen in CT. This figure was previously presented by Jerban et al. (61). Reprinting permission is granted through Rightslink system. The figure is modified for presentation purposes. Minor modifications were performed for presentation purposes. 3D UTE sequence parameters: FOV = 14×14×14 cm3; voxel size = 0.7×0.7×5 mm3, scan time » 3.2 mins. 3D IR-UTE sequence parameters: FOV = 14×14×14 cm3; voxel size = 0.7×0.7×5 mm3, TR/TI = 300/110 ms, scan time » 3.5 mins.

Conventional MRI sequences generally visualize the bone tissue with a signal void surrounded by bright signal from adjacent soft tissues. Pore water may compose up to a quarter of the bone volume and possesses a short T2* and relatively long T2 (up to 100 ms) (10, 13–15). Therefore, some conventional fast spin echo (FSE) (27) and short echo time (STE) c sequences have the potential to image pore water in cortical bone, even though clinical gradient recalled echo (GRE) sequences are not capable of pore water imaging, as shown in **Figure 2** (**Figures 2A, B**) and **Figure 3A**. Consequently, FSE and STE techniques might be useful in qualitative imaging of highly porous cortical bone sites, which can be found more in elderly cohorts. Nevertheless, qualitative imaging of cortical bone with low porosity requires more advanced MR imaging techniques.

## UTE MR Bone Imaging

UTE MRI sequences with nominal TEs around tens of microseconds or less can detect signals from both bound water (T2* ≈ 300 µs) and pore water (T2* > 1,000 µs) pools in cortical bone, as demonstrated in **Figure 2** (**Figures 2C, D**) (6, 13). Collagen protons have extremely short T2*s (T2* on the order of several microseconds) and are not detectable, even when using UTE MRI sequences. The effective TEs in UTE are significantly longer than the T2*s of collagen backbone protons (≈ 10 µs) because of the use of a relatively long RF excitation pulse as well as time-consuming ramp sampling (8, 10, 15, 29). UTE MRI imaging results in high *in vivo* signal of bone, but it is still lower than the signal from surrounding soft tissues, which in turn leads to relatively low contrast as demonstrated in **Figure 3B**. Improving the UTE contrast in bone imaging requires fat suppression in trabecular bone sites while fat and soft tissue suppression in cortical bone sites. Moreover, pore water suppression can improve the cortical bone UTE contrast by imaging only the bound water.

## IR-UTE MR Imaging of Cortical Bone

Adiabatic inversion recovery (IR) preparation pulses have been suggested in different studies to suppress long T2 tissue components and increase the contrast in UTE MR imaging of cortical bone (**Figure 1**). The bound water component in cortical bone can be selectively imaged with both 2D and 3D IR-UTE sequences. In both cases, a relatively long adiabatic IR pulse (e.g., Silver-Hoult pulse, 8.64 ms in duration) is employed to invert the longitudinal magnetizations of first, long T2 water (e.g., free water components of cortical bone, and water in muscle) and second, fat in cortical bone and bone marrow (25, 30, 32–34). However, the simultaneous suppression of pore water, fat, and muscle is highly challenging due to the significant differences between MR properties of these three proton pools. Thus, IR-UTE based imaging of bound water may coexist with some level of pore water, fat, and muscle contamination. The 2D or 3D UTE data acquisition starts at an inversion time (TI) designed to allow the inverted free water and fat longitudinal magnetizations to be at or close to the null point (**Figure 1**) (30). **Figure 2** shows a comparison between conventional MRI (FSE and GRE), UTE, and IR-UTE sequences performed on a piece of anterior tibial cortex imaged in axial plane. The conventional FSE sequence produces higher signal in cortical bone compared with the GRE sequence, though both techniques only detect signal from pore water residing in Haversian canals. The UTE sequence can detect free water in the pores (high signal with fine structure) and bound water (uniform background signal). The signal of the fine structure will disappear using the IR-UTE sequence which suppresses the free water signal while the uniform background signal from bound water remains.

**Figure 3** shows tibial and fibular midshaft bone in a healthy young volunteer imaged with GRE, UTE and IR-UTE sequences. In UTE MRI, bone shows as high signal compared with conventional MRI technique, but as low signal compared with surrounding tissues such as marrow fat and muscle. The IR-UTE technique greatly suppresses signals from both the marrow fat and muscle, resulting in visualization of cortical bone with a CT-like contrast that can be used for qualitative bone evaluation.

Adiabatic inversion recovery (AIR) UTE (AIR-UTE) (35, 36) is an alternative abbreviation used for abovementioned IR-UTE technique by other groups. AIR-UTE is used to visualize bound water in cortical bone and provide a qualitative image of cortical bone structure.

**Figure 4:**
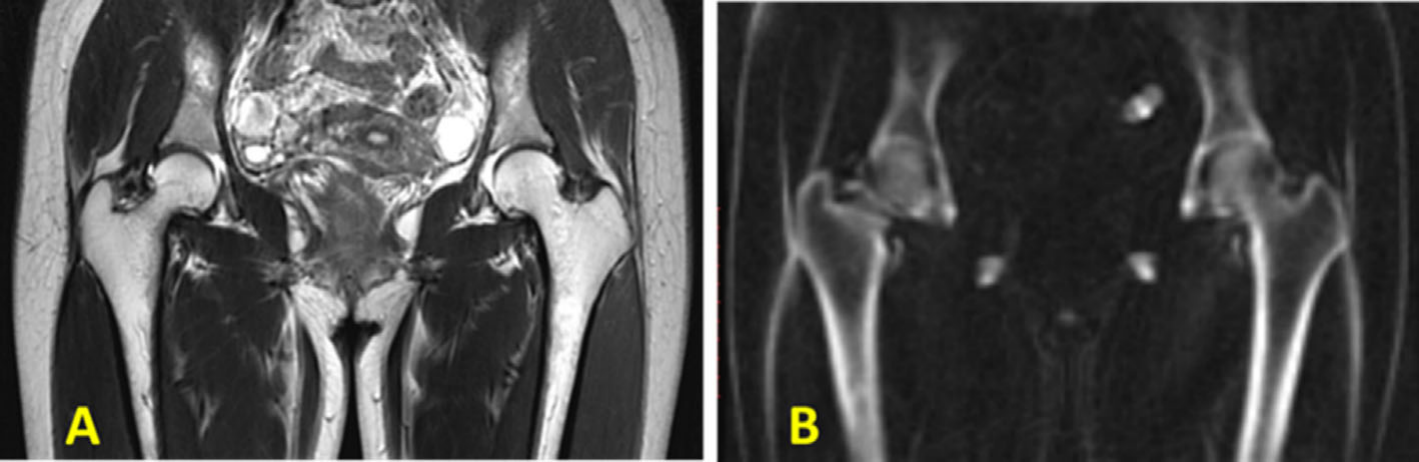
In vivo imaging of the hip of a 24‐year‐old female volunteer with a **(A)** clinical 2D T2‐weighted FSE and **(B)** 3D IR‐UTE Cones sequences. Soft tissues are well‐suppressed in the 3D IR‐UTE Cones image, while they are bright in the clinical T2‐FSE images. This figure was previously presented by Ma et al. (31). Reprinting permission is granted through Rightslink system. The figure is modified for presentation purposes. Minor modifications were performed for presentation purposes. 3D IR-UTE sequence parameters: FOV = 38 cm^3^ × 38 cm^3^ × 20 cm^3^; voxel size = 2.4 mm^3^ × 2.4 mm^3^ × 5 mm^3^, TR/TI = 150/64 ms, and scan time ≈ 9.5 min.

The IR-UTE-based qualitative bone imaging sequence has been applied to different bone sites *in vivo* such as tibia and fibula (36–38), radius (36), hip (34), and shoulder (39); however, the contrast and image quality depend on the anatomical location, coil quality and size, B0 and B1 homogeneity, bone thickness, image resolution, and slice thickness. These factors affect the proper visualization of the bone structure, signal to noise ratio (SNR), suppression uniformity, and image artifacts. Bone sites in axial skeleton or deeply located in the body such as spine and hip are more difficult to be scanned compared with tibia and radius in peripheral bone sites. Specifically, sophisticated thin bone structure requires high resolution imaging which is highly sensitive to motion. Larger required coils result in greater B1 inhomogeneity and non-uniform suppression. **Figure 4** illustrates coronal images of the hip and femoral head of a healthy young volunteer using 2D FSE and 3D IR-UTE Cones sequences (31).

## Dual-IR-UTE Imaging of Cortical Bone

**Figure 5:**
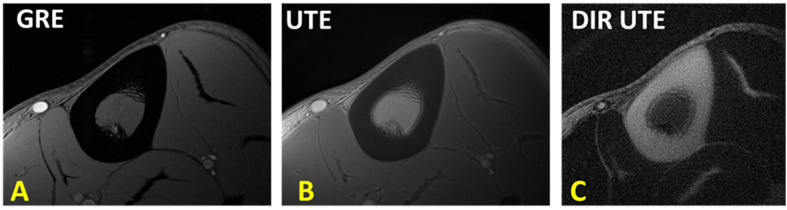
The tibial midshaft of a healthy young volunteer imaged with **(A)** GRE, **(B)** UTE and dual adiabatic inversion recovery UTE (Dual-IR-UTE) sequences. The Dual-IR-UTE image **(C)** selectively suppresses signal from fat and muscle, and which creates high contrast for cortical bone. This figure was previously presented by Du et al. (33). Reprinting permission is granted through Rightslink system. The figure is modified for presentation purposes. Minor modifications were performed for presentation purposes. 2D UTE sequence parameters: FOV = 10 cm^2^ × 10 cm^2^; voxel size = 0.2 cm^2^ × 0.2 cm^2^ and scan time ≈ 3 min. 3D Dual-IR-UTE sequence parameters: FOV = 10 cm^2^ × 10 cm^2^; voxel size = 0.2×0.2, TR/TI1/TI2 = 300/140/110 ms, and scan time ≈ 3 min.

Dual-adiabatic inversion recovery (Dual-IR) pulses can also be employed to invert and null signals from long T2 water and fat, respectively (33). Dual-IR followed by selective UTE imaging can detect bound water in cortical bone and provide qualitative bone evaluations. In this approach, two successive long adiabatic inversion pulses are employed to invert the longitudinal magnetization of long T2 water and long T2 fat, respectively (33, 40, 41). The pulse sequence diagram for Dual-IR-UTE is similar to IR-UTE sequence hovers with an additional inversion recovery pulse (**Figure 1**). As a result of a significant transverse relaxation of cortical bone magnetization with short T2 during the long adiabatic inversion process, its longitudinal magnetization cannot be inverted (62). The UTE MRI data acquisition begins after the first delay time (TI1), which is required for the nulling of the inverted long T2 water magnetization, and the second delay time (TI2), which is required for the nulling of the inverted fat magnetization. **Figure 5** shows representative images of the left distal tibia of a volunteer using clinical 2D GRE, UTE and Dual-IR-UTE techniques. Cortical bone is visualized with a void signal using the 2D GRE pulse sequence, however with a poor contrast using the basic UTE pulse sequence because of the significant signals acquired from surrounding muscle and fat. The Dual-IR-UTE sequence suppresses long T2 water signals, including those from muscle and free water in bone, as well as from marrow fat, and displays bound water in cortical bone with high signal and contrast. Dual-IR UTE can be applied to different body regions. However, this technique only works well for simultaneous water and fat suppression when the frequency offset due to the B0 inhomogeneity is less than half of the bandwidth of the used adiabatic IR pulse.

## Double-Inversion Recovery UTE (Double-IR-UTE)

**Figure 6:**
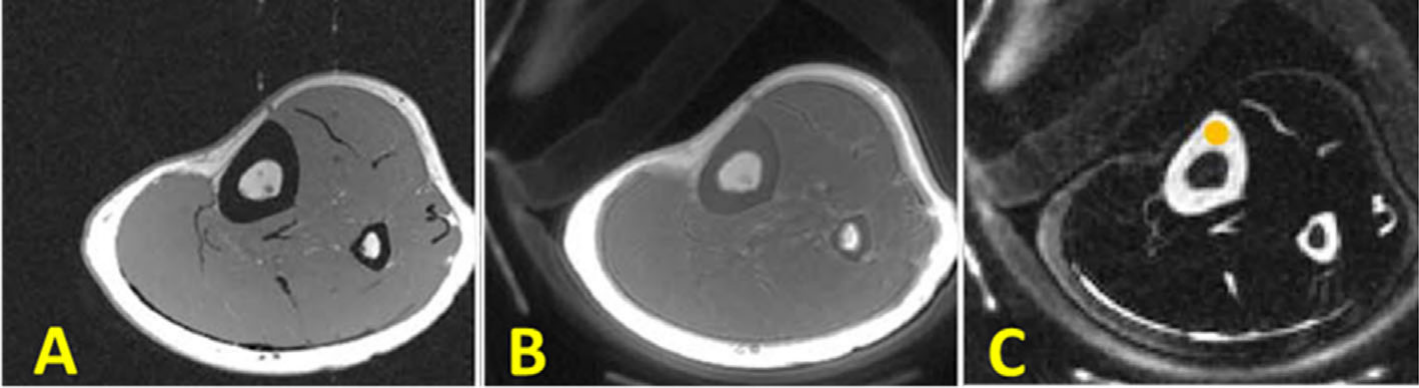
In vivo imaging of the lower leg of young healthy volunteer using **(A)** clinical 3D GRE sequence (no signal in cortical bone), **(B)** the conventional 3D UTE sequence (detects signal in cortical bone signal but with low contrast), and **(C)** the 3D Double-IR-UTE sequence which shows simultaneous suppression of muscle and marrow fat, providing higher contrast between cortical bone and surrounding soft tissue. This figure was previously presented by Ma et al. (42). Reprinting permission is granted through Rightslink system. The figure is modified for presentation purposes. Minor modifications were performed for presentation purposes. 3D UTE sequence parameters: FOV = 14 cm^3^ × 14 cm^3^ × 12 cm^3^; voxel size = 0.55 mm^3^ × 0.55 mm^3^ × 6 mm^3^, and scan time ≈ 1.3 min. 3D Double-IR-UTE sequence parameters: FOV = 14×14×12 cm3; voxel size = 1.1 mm^3^ × 1.1 mm^3^ × 6 mm^3^, TR/TI1/TI2 = 200/100/45 ms, and scan time ≈ 2.9 min.

Double inversion recovery (Double-IR) UTE sequence also employs two adiabatic inversion pulses to invert and null signals from long T2 tissues (42). Despite Dual-IR-UTE sequence, Double-IR-UTE utilizes two identical adiabatic inversion pulses. The center frequency of these identical pulses are located at the water peak while their spectral widths are broad enough to cover both water and fat frequencies (42). These two adiabatic inversion pulses are applied in sequence with two different inversion times (TI1 and TI2) in order to invert and null the longitudinal magnetizations of long T2 muscle and fat. Similar to Dual-IR-UTE technique, due to the significant transverse relaxation of the cortical bone magnetization during the long adiabatic inversion pluses, its longitudinal magnetization cannot be inverted. The pulse sequence diagram for Double-IR-UTE is similar to IR-UTE sequence hovers with an additional inversion recovery pulse (**Figure 1**). **Figure 6** shows representative images of the left distal tibia of a volunteer using clinical 3D GRE, UTE and Double-IR-UTE techniques (42). The Double-IR-UTE demonstrated cortical bone with high signal and contrast as the signal from long T2 water and fat were suppressed robustly. Notably, bone was visualized with a signal void using the 3D GRE sequence, and with a poor contrast using the conventional UTE sequence.

All the IR-UTE-based techniques (IR-UTE, Dual-IR-UTE, Double-IR-UTE, and AIR-UTE) appear to provide a uniform suppression of long T2 water and fat signals, which in turn provides a good qualitative image of the targeted bone. This is because adiabatic IR pulses are relatively insensitive to B1 and B0 inhomogeneities (41, 62).

To the best of our knowledge, there is no detailed comparison study for these techniques. Based on our experience, IR-UTE sequence is more time-efficient than both Dual- and Double-IR UTE sequences. IR- and Double-IR UTE sequences are much less sensitive to the B0 inhomogeneity than Dual-IR UTE sequence. Dual-IR sequence provides a better SNR compared with Double-IR UTE sequence. It would be interesting to perform a future study to compare all these techniques regarding the performance of SNR, CNR, and the corresponding scan efficiency. The comparisons presented in **Table 1** have been prepared based on the practical experience of the authors.

## Fat Suppression UTE

**Figure 7:**
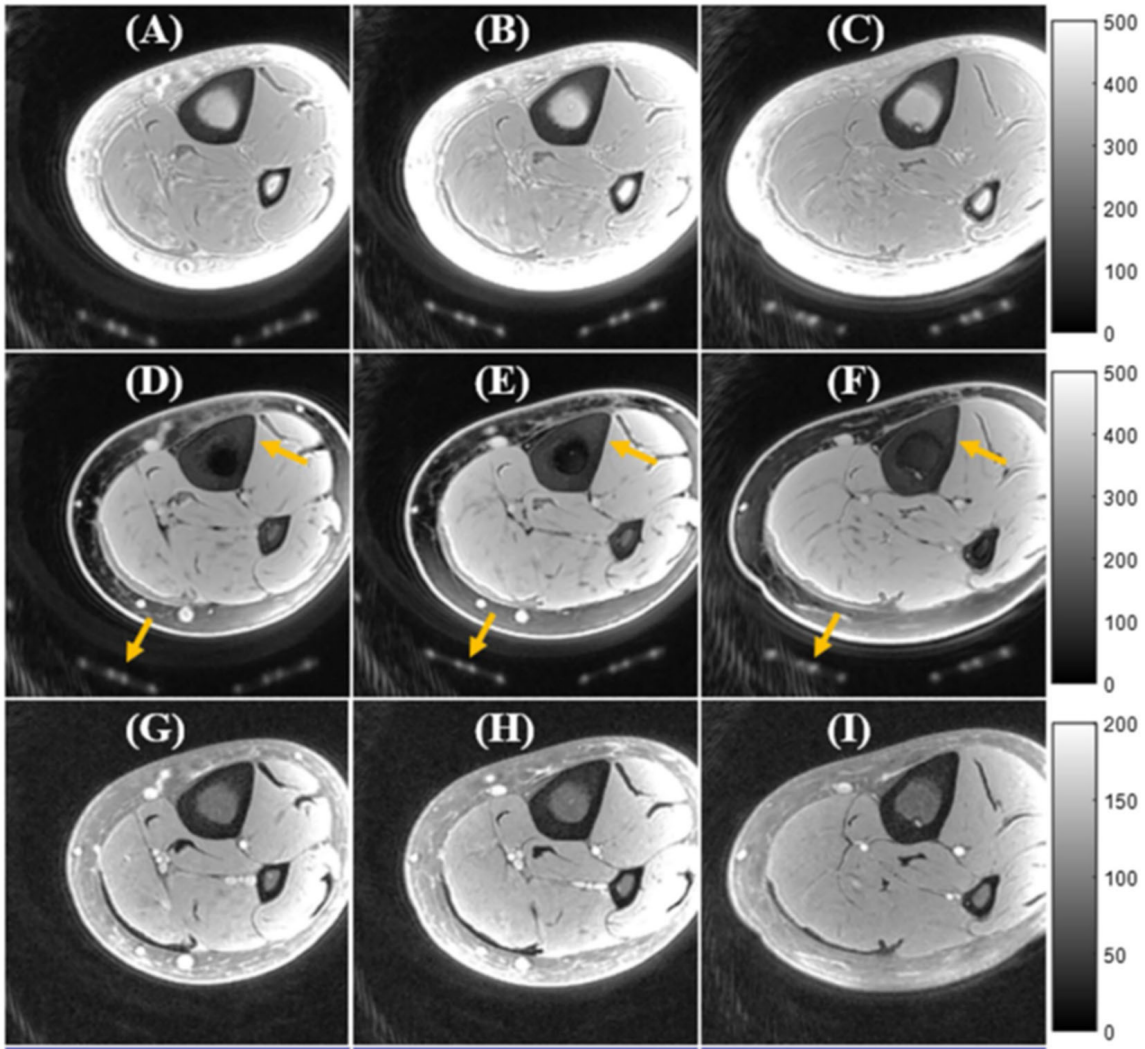
In vivo UTE Cones imaging of tibial midshaft of young healthy volunteer using **(A–C)** a single hard pulse excitation (basic UTE), **(D–F)** the soft-hard water excitation pulse, and (G-I) the conventional FatSat module. UTE images are presented in three representative slices. Fat was well suppressed by both the soft-hard pulse and the FatSat module. The cortical bone and coil elements (indicated by yellow arrows in **D–F**) were much better preserved in the soft-hard excitation images **(D–F)** compared with FatSat images **(G–I)**. This figure was previously presented by Ma et al. (43). The reprinting permission is granted through Rightslink system. The figure is modified for presentation purposes. Minor modifications were performed for presentation purposes. 3D UTE sequence parameters: FOV = 12 cm^3^ × 12 cm^3^ × 16 cm^3^; voxel size = 0.63 mm^3^ × 0.63 mm^3^ × 2 mm^3^, and scan time ≈ 3.4 min.

Human bone exists in combination with bone marrow which possess high percentage of fat. The fat presence results in chemical shifts and average signal oscillation observed in the multi-echo MRI in T2 fitting analyses (63). Fat suppression techniques can be used to remove fat signal contamination in bone assessment and to improve the bone contrast in UTE-MRI. Chemical shift fat saturation (FatSat), soft-hard water excitation, and single point Dixon methods have been employed to suppress fat in UTE bone imaging (43, 44). FatSat is widely used in clinical MR sequences, however, it is not suitable for bone imaging due to the strong signal saturation of the wide spectrum band of bone. The soft-hard pulse has been proposed to overcome the signal attenuation effect *via* utilizing a low power soft-pulse for fat excitation in the opposite direction of the following hard pulse (43). **Figure 7** shows a comparison between tibial and fibular bone image contrast *in vivo* obtained using basic UTE, FatSat, and the soft-hard water excitation techniques (43). The fat signal could be suppressed well using both the soft-hard pulse and the FatSat module in the three illustrated slices. However, the cortical bone signal (indicated by yellow arrow) were much better preserved in the soft-hard excitation images (**Figures 7D–F**) (43). Single-point Dixon method is a postprocessing method to separate water and fat signals from a dual-echo UTE acquisition (44). The calculated water and fat maps can then be used to suppress fat in the UTE image.

## UTE With Rescaled Echo Subtraction (UTE-RS)

**Figure 8:**
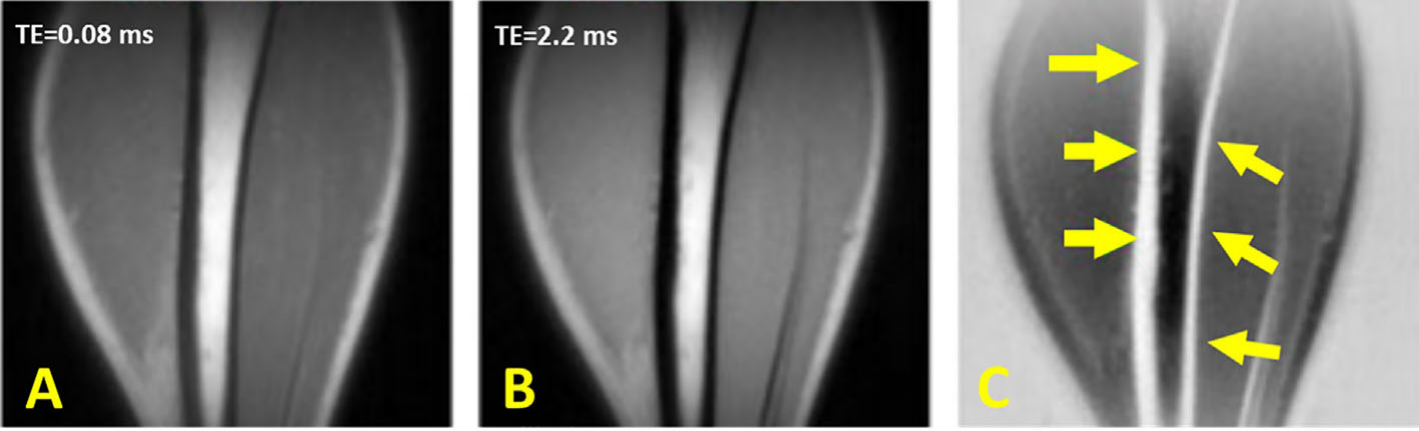
Dual-echo 3D UTE imaging of the tibial cortex of a healthy young volunteer in coronal plane with **(A)** TE = 8 μs and **(B)** TE = 2.2 ms. **(C)** Subtraction of the second echo from the first echo image rescaled down by a factor of 0.4. This figure was previously presented by Du et al. (46). The reprinting permission is granted through Rightslink system. The figure is modified for presentation purposes. Minor modifications were performed for presentation purposes. 3D UTE sequence parameters: FOV = 24 × 24 cm^3^ × 24 cm^3^; voxel size = 0.8 mm^3^ × 0.8 mm^3^× 0.8 mm^3^, TEs = 0.008/2.2 ms, and scan time ≈ 5.5 min.

UTE with rescaled echo subtraction (UTE-RS) can provide qualitative imaging of the cortical bone (46, 47). In UTE-RS, the free induction decay (FID) image is scaled down so that signals from muscle and fat become lower than those from the second echo (46). In the subtraction image, signals from muscle and fat are negative, whereas those from short-T2 species are positive, separating them from air which has a signal intensity fluctuating around zero (46). The UTE-RS technique can be efficient in creating high positive contrast for short T2 species such as cortical bone. Regular unscaled echo subtraction may reduce bone contrast in this situation. Performing UTE-RS in trabecular bone sites would be challenging due to the fat presence in bone marrow and signal oscillation. Rescaled echo subtraction method has been also used with ZTE technique (64). **Figure 8** shows the tibial cortex of a healthy young volunteer imaged in the coronal plane using the UTE-RS technique.

## Water- and fat-suppressed proton projection imaging (WASPI) of cortical bone

**Figure 9:**
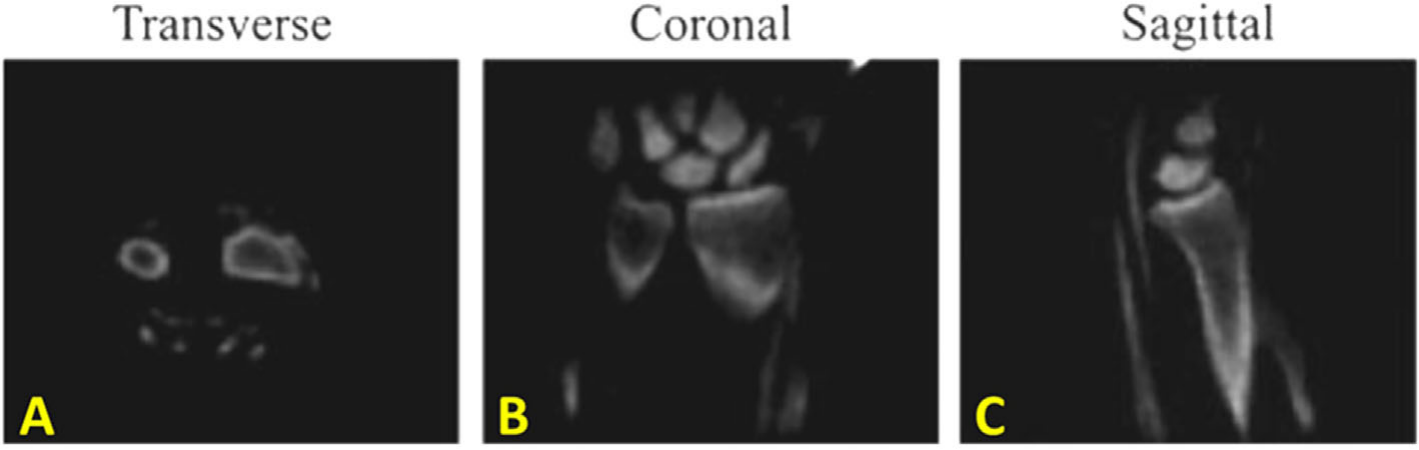
In vivo water- and fat-suppressed proton projection imaging (WASPI) of the wrist joint of a healthy volunteer in **(A)** transverse, **(B)** coronal and **(C)** sagittal slices from the 3D WASPI image dataset. This figure was previously presented by Wu et al. (51). Reprinting permission is granted through Rightslink system. The figure is modified for presentation purposes. Minor modifications were performed for presentation purposes. 3D WASPI sequence parameters: FOV = 12 cm^3^ × 12 cm^3^ × 12 cm^3^; voxel size = 0.9 mm^3^ × 0.9 mm^3^ × 0.9 mm^3^, and scan time ≈ 18 min.

Water- and fat-suppressed proton projection MRI (WASPI) is another MRI sequence developed for selective imaging of bone water bound to the organic matrix (48–50). In this technique, two long-duration, yet low-power, rectangular RF pulses are used to selectively saturate signals from long T2 water and fat. Since bound water has a short T2*, it will remain largely unsaturated and provide qualitative imaging of bound water in bone. **Figure 9** shows bone WASPI images in the wrist joint of a healthy volunteer in transverse, coronal and sagittal planes (51).

## ZTE Bone Imaging

**Figure 10:**
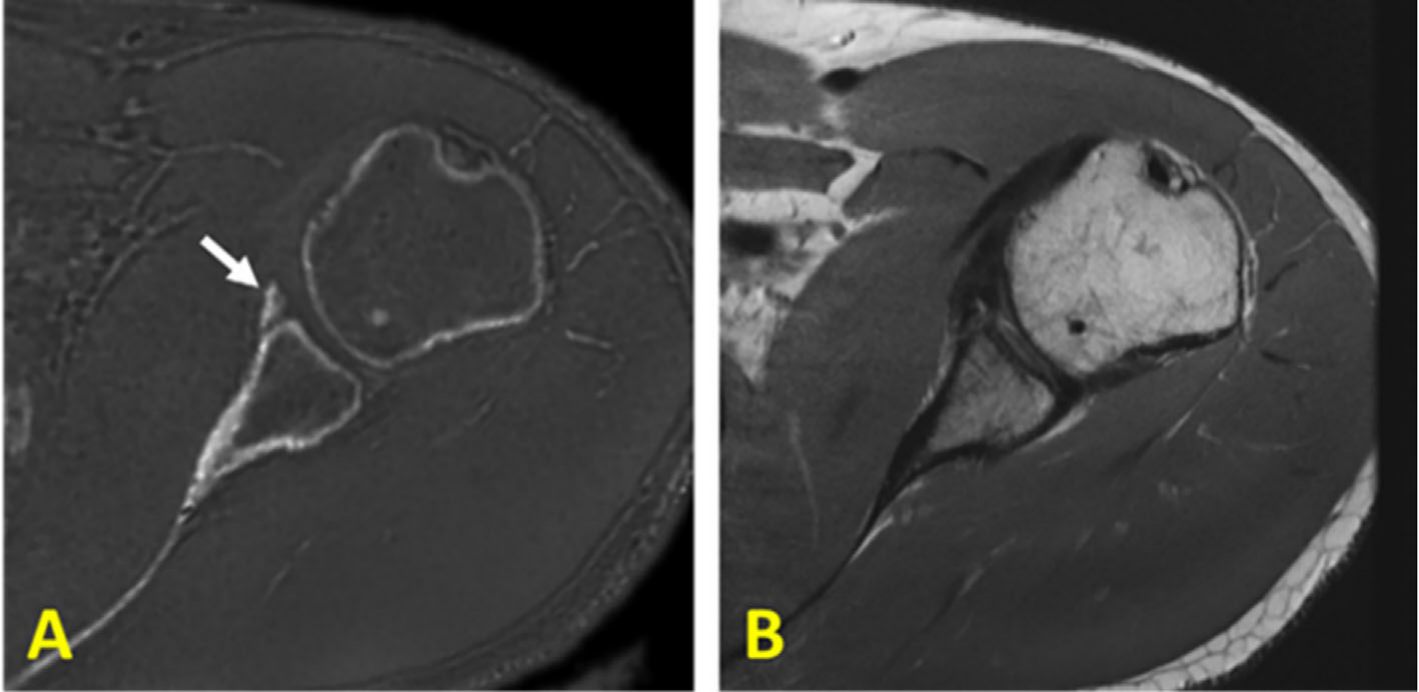
**(A)** Zero echo time (ZTE)-based and **(B)** proton-density-weighted FSE MR imaging of the shoulder of a young symptomatic patient. The CT-like ZTE-based image **(A)** is obtained after bias-correction and inverse-logarithmic rescaling (52). Qualitative bone imaging by ZTE shows the bone fragment (indicated with arrow), which is not clear in FSE image. This figure was previously presented by Breighner et al. (52). Reprinting permission is granted from Radiology journal (RSNA). The figure is modified for presentation purposes. Minor modifications were performed for presentation purposes. 3D ZTE sequence parameters: FOV = 28 cm^3^ × 28 cm^3^ × 28 cm^3^; voxel size = 0.87 mm^3^ × 0.87 mm^3^ × 1.5 mm^3^, and scan time ≈ 5 min.

**Figure 11:**
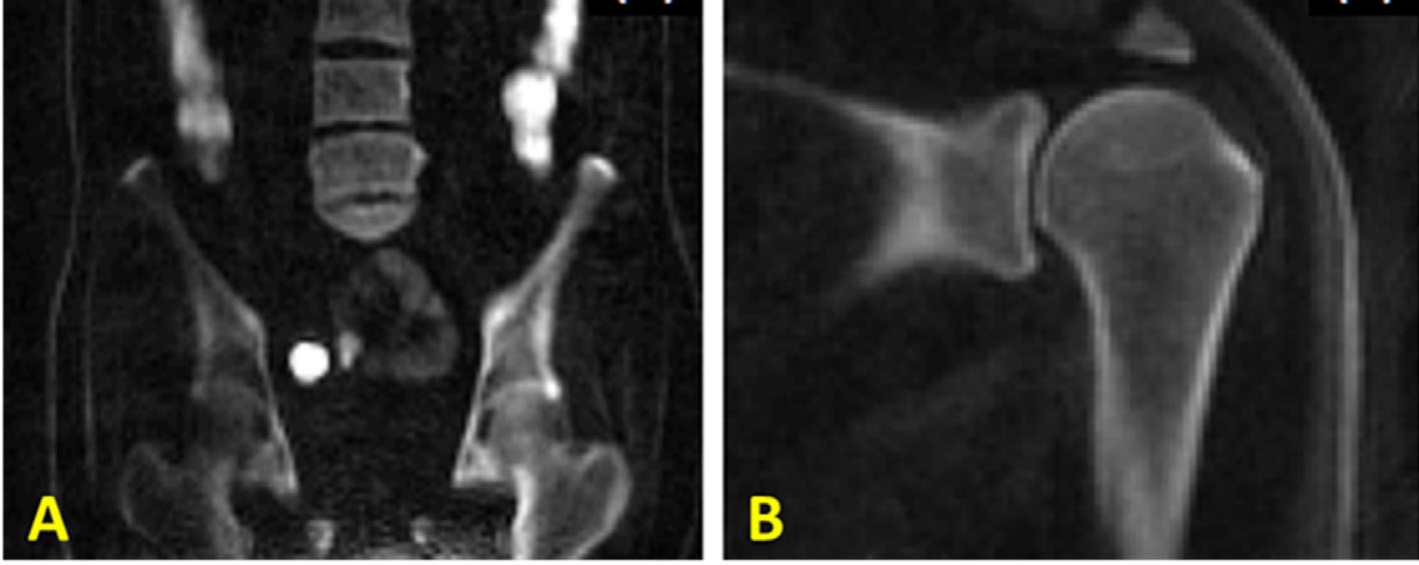
3D IR-UTE-Cones imaging of **(A)** the femur/spine and **(B)** the shoulder sample in the coronal plane, demonstrating high contrast imaging of cortical and trabecular bone at various sites in the body using a clinical whole-body 3T scanner. 3D IR-UTE sequence parameters for femur/spine: FOV = 45×45×20.8 cm3; voxel size = 2.5×2.5×4 mm3, TR/TI = 183/78 ms, and scan time » 10 mins. 3D IR-UTE sequence parameters for shoulder: FOV = 20× 20 × 10 cm3; voxel size = 0.8×0.8×2 mm3, TR/TI = 140/61 ms, and scan time » 6 mins. This figure is original and based on data from (31). A version of this figure has been presented before in ISMRM 2019 conference (poster 3754).

An alternative approach for bone imaging is the zero echo time (ZTE) sequence, which employs a short rectangular excitation pulse during the fully ramped up readout gradient, followed by fast radial sampling (52, 53). Alternatively, frequency-modulated pulse with interleaved transmit-receive operation, can also be used for bone imaging which is known as sweep imaging with Fourier transformation (SWIFT) (54). Eliminating the rapid gradient switching between TR intervals results in the decreased acoustic noises during scans and in the reduced eddy current artifacts (5). These sequences have the potential to image both bound water and pore water in bone. These sequences suffer from a gap of data at the center of k-space as a result of a dead time caused by the finite RF pulse, transmit-receive switching, and digital bandpass filtering (53). The missing data in the ZTE technique can be compensated *via* oversampled acquisition and mathematical reconstruction (53). The ZTE data gap can be also filled using a Cartesian single-point imaging technique, which is known as pointwise encoding time reduction with radial acquisition (PETRA) (55). **Figure 10** shows representative postprocessed ZTE and conventional FSE images of the shoulder of a young symptomatic patient. CT-like ZTE-based image (**Figure 11A**) was obtained after bias-correction and inverse-logarithmic rescaling (52). The qualitative ZTE bone image clearly visualizes the bone fragmentation, which is not clear in FSE image (52). To improve the image contrast ZTE techniques has been also used with inversion recovery preparation which suppress long-T2 signal (56).

## IR-UTE Imaging of Trabecular Bone

Direct MR imaging of trabecular bone is technically challenging due to its fast signal decay, the fat presence in bone marrow, and local field inhomogeneities that are caused by the trabecular bone structure (24, 45, 65). Recently, UTE sequences and their variants (e.g., WASPI and ZTE sequences) have been developed to directly acquire the signals of phosphorus (66–68) or hydrogen proton (45, 69, 70) in trabecular bone. Such techniques have been used successfully for phosphorus density and its relaxation times assessment *in vivo* for both cortical and trabecular bone (66–68). However, further optimization of these techniques for translation to clinical investigations is limited because the required hardware for phosphorus imaging is not available in most clinical scanners.

Direct hydrogen proton imaging of trabecular bone using WASPI and fat-suppressed UTE techniques have also been investigated recently (45, 69, 70). Suppressing signals from long T2 components in bone marrow is crucial in order to create a high contrast for bone in trabecular sites. WASPI technique uses two hard pulses with narrow frequency bands in order to selectively excite water and fat signals. Following the two hard pulses, strong gradient crushers are used to saturate water and fat signals before data acquisition (48). Wurnig et al. employed the UTE sequence to measure T2* of trabecular bone samples, with a SPIR (spectral pre-saturation with inversion recovery) module to suppress marrow fat (45). These two techniques show promising results for trabecular bone, though they are both sensitive to B1 and B0 inhomogeneities, posing clinical challenges for *in vivo* imaging of trabecular bone in the hip and spine.

To resolve these issues, a broadband adiabatic inversion recovery prepared 3D UTE Cones (3D IR-UTE-Cones) sequence has been proposed for direct volumetric imaging of bone by simultaneous water and fat signal suppression (71) which later being used for direct trabecular bone imaging (31). The combination of a short repetition time (TR) and inversion time (TI) is used to achieve robust suppression of different long T2 tissues with a range of T1s. Employing an adiabatic full passage (AFP) pulse sequence with a relatively wide bandwidth (~1.5 kHz), the proposed IR preparation is approximately insensitive to the inhomogeneities of B1 and B0 (72). **Figure 11** shows high contrast imaging of trabecular bone in the shoulder, femoral head and neck, as well as the spine with the 3D IR-UTE-Cones sequence (31).

## Conclusions

MRI-based qualitative imaging of different water compartments in bone has drawn great interest among musculoskeletal radiologists and orthopedic researchers. These techniques provide simultaneous assessment of bone and the surrounding soft tissues, while avoiding patient exposure to ionizing radiation. Since conventional clinical MRI techniques fail to detect signal from bone, here are several qualitative MR techniques currently being developed for assessment of cortical bone. Such techniques include UTE MRI, which detects signal from both bound water and pore water in bone, but with low contrast due to much higher signals from the surrounding soft tissues such as muscle and bone marrow fat. IR-UTE-based sequences provide efficient suppression of long T2 tissues, allowing bound water imaging with CT-like bone contrast. Other techniques, such as UTE-RS, WASPI, ZTE and SWIFT also have potential for qualitative bone imaging. Some of the described techniques can be used for quantitative bone imaging and for microstructural evaluations.

This study would be greatly improved if the presented MRI images would be acquired from subjects with bone diseases or injuries resulting in a better comparison between presented techniques. However, most published results in the literature only have covered healthy bone images. Comparison between the presented techniques in **Table 1** is largely based on practical experience of the authors. Particularly, the scan time efficiency was ranked semi-qualitatively, and is affected by acquisition parameters (e.g., undersampled acquisition, extended sampling, etc.) and reconstruction algorithms.

## Author Contributions

All authors contributed to the article and approved the submitted version.

## Funding

The authors acknowledge grant support from NIH (R01AR068987, R01AR075825, R01AR062581, R21AR075851) and VA Clinical Science and Rehabilitation R&D Awards (I01CX001388 and I01RX002604).

## Conflict of Interest

The authors declare that the research was conducted in the absence of any commercial or financial relationships that could be construed as a potential conflict of interest.
